# Subretinal Cysticercosis in a Challenging Case: A Case Report

**DOI:** 10.31729/jnma.6732

**Published:** 2021-10-31

**Authors:** Ruchi Shrestha, Ritesh Kumar Shah, Purushottam Joshi

**Affiliations:** 1 Department of Vitreoretina, Reiyukai Eiko Masunaga Eye Hospital, Banepa, Kavre, Nepal; 2 Department of Vitreoretina, Mechi Netralaya, Kakarvitta, Jhapa, Nepal; 3 Department of Vitreoretina, Mechi Eye Hospital, Birtamod, Jhapa, Nepal

**Keywords:** *cysticercosis*, *subretinal*, *vitrectomy*

## Abstract

Ocular cysticercosis occurs rarely and may involve various parts of the eye including subretinal space. We report a case of a 42 years-old female with diminution of vision in the right eye for one month and no vision in the left eye for 10 years. Best corrected visual acuity in the right eye was 5/60. Fundus examination showed whitish round elevated cystic mass temporal to the macula. It was confirmed as a subretinal cyst by Brightness scan and Magnetic Resonance Imaging. The subretinal cyst was removed in toto by pars plana vitrectomy followed by histopathological examination of the cyst which confirmed the diagnosis of cysticercosis. This case report highlights the importance of early diagnosis of subretinal cysticercosis which could threaten the vision in a one-eyed patient. Pars plana vitrectomy could be an effective method for subretinal cyst removal in toto even in a challenging case.

## INTRODUCTION

Cysticercosis may involve various parts of the body including the eye (13% to 46%), subcutaneous tissue (24.5%), and the brain (3. 6 %).^1^ The possible encystment sites in the eye are subretinal space, vitreous, subconjunctival space, anterior chamber, recti muscles, lids, lacrimal gland, and lens.^1^

The cysticercus parasite enters the subretinal space through the posterior ciliary arteries and may migrate into the vitreous cavity.^2^ Ocular cysticercosis occurs rarely. The diagnosis is very important as untreated intraocular cysticercosis will lead to blindness.^3^ We present a challenging case of subretinal cysticercosis removed in toto by pars plana vitrectomy in a one-eyed patient.

## CASE REPORT

A 42 years-old female came to the out-patient department with the chief complaint of diminution of vision in the right eye for 1 month. Diminution of vision was gradual in onset, painless and progressive. It was associated with floaters in the right eye. The patient had no vision in the left eye for 10 years. The patient had a past history of left-sided hemiplegia and Multiple Sclerosis for ten years. There was no history of other systemic diseases like Diabetes Mellitus and Hypertension. The patient was vegetarian by diet for 2 years but had a history of eating pork meat 2 years back. There was no history of contact with pets (Cat or Dog). There was no significant family and psychosocial history.

**Figure 1:**
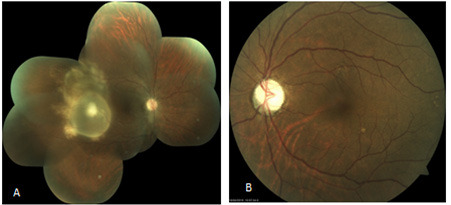
(A) Fundus Photo of right eye showing subretinal cyst just temporal to macula with whitish dense scolex like material. (B) Fundus photo of left eye showing pale disc with dull foveal reflex.

The Best Corrected Visual Acuity (BCVA) at presentation was 5/60 in the right eye and perception of light (PL) only in the left eye. The intraocular pressure (IOP) was 11 mmHg in the right eye and 15mmHg in the left eye. The conjunctiva and cornea were healthy. There was Rapid Afferent Pupillary Defect (RAPD) in the left eye. There were 2+ anterior chamber reactions and 2+ vitreous chamber reactions in the right eye. The lens was clear in both eyes. The media was clear in both eyes. The posterior segment in the left eye had a pale disc with no significant increase in cup size suggestive of optic atrophy. The posterior segment in the right eye had a cup to disc ratio of 0.3 with a healthy neuroretinal rim. There was a whitish round elevated cystic mass seen 2 disc diameter temporal to the macula and about 3-4 disc diameter in size with posterior vitreous detachment (Figure 1).

**Figure 2:**
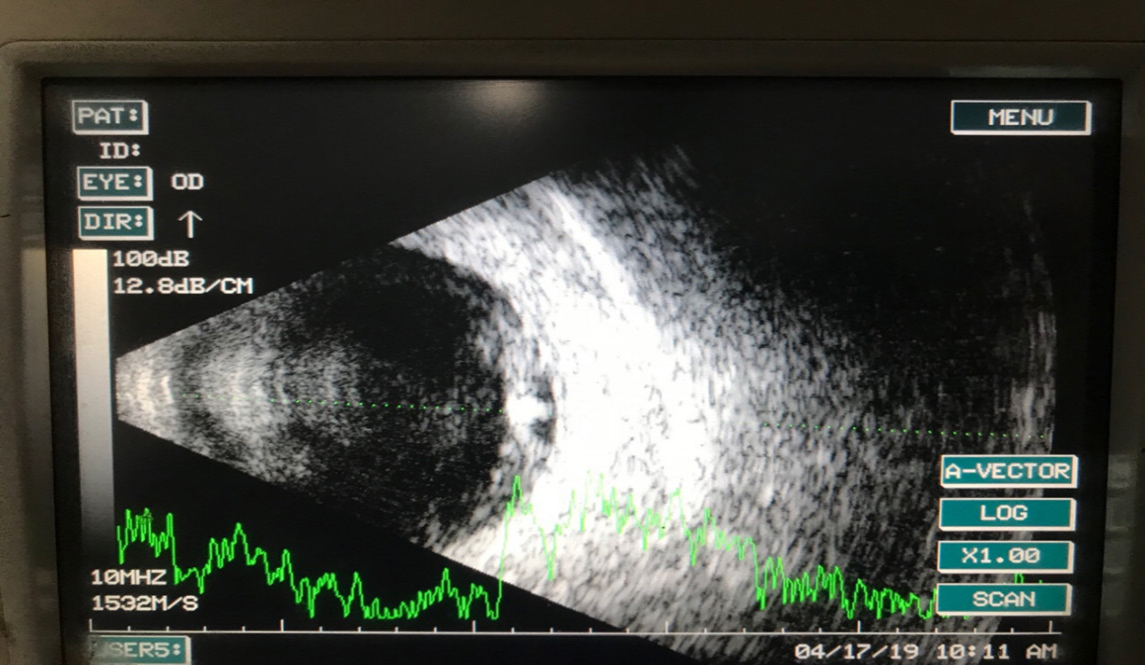
Brightness amplitude scan of right eye showing dome shaped elevation with a high reflective elevation at Inferotemporal quadrant suggestive of scolex.

The cyst had a dense white material that was suspected to be a cysticercoid cyst with a scolex within it. The patient was sent for investigation. Brightness scan (B-scan) showed a dome-shaped elevation with a high reflective elevation at the inferotemporal quadrant suggestive of the scolex (Figure 2).

Magnetic resonance imaging (MRI) showed small nodular hypointense foci on SW1 images in both the cerebral hemispheres including the right thalamo-basal ganglia and left cerebellum suggestive of chronic hemorrhagic lesions. The lesions represented calcific foci with the probability of calcified cysticerci. The left optic nerve was smaller in the left eye suggestive of optic atrophy. Unfortunately, we couldn't provide the images of the MRI scan and just got the reports. Blood investigations were sent. The complete blood count (CBC) was normal. The serology, Rheumatoid Arthritis (RA) Factor, and Tuberculosis (TB) antibody were negative. The stool routine microscopy was normal with no cysts or parasites seen. A provisional diagnosis of right eye subretinal cyst most likely due to cysticerci and left eye optic atrophy secondary to multiple sclerosis was made. It was very challenging to think of surgery as the patient was one-eyed but there was the risk of spilling the cyst contents to threaten the vision. The subretinal cyst was threatening the vision in the good eye so the surgery was planned as soon as possible.

**Figure 3:**
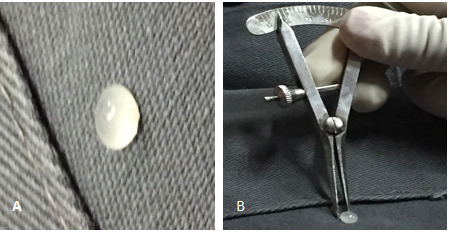
(A) Subretinal cyst in toto after pars plana vitrectomy with dense white scolex like material within it. (B) Cyst about 5mm in size measured by callipers.

The patient underwent right eye pars plana vitrectomy with subretinal cyst removal, endo laser, and silicon oil insertion. The subretinal cyst was surgically removed and sent for histopathological examination. Grossly the cyst was cystic in nature with whitish material within suggestive of cysticerci with scolex (Figure 3).

**Figure 4:**
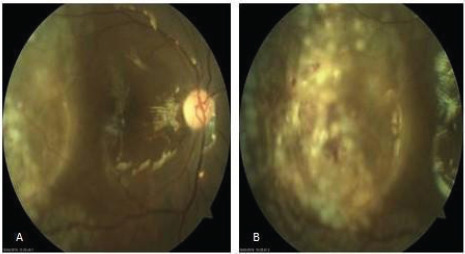
(A) Fundus photo of right eye on first postoperative day with silicone oil in situ. (B) Fundus photo of the temporal retina where the cyst was removed with whitish laser marks.

The Best Corrected Visual Acuity in the right eye was 1/60 on the first post-operative day. The intraocular pressure was 10 mmHg. There were few keratic precipitates (KPs) in the corneal endothelium. The anterior chamber reaction was 3+ cells. The fundus examination on the first postoperative day showed a well-attached retina, laser marks, and silicone oil in situ (Figure 4 a,b).

The Best Corrected Visual Acuity was 6/36 with +6.00 Dioptre spherical lens in the right eye at two weeks follow up. Fundus examination at 2 weeks showed a well-attached retina with laser marks around the cyst area and silicon oil in situ.

**Figure 5:**
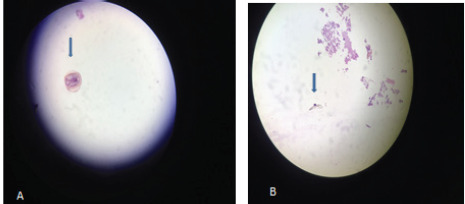
(A) Histopathology slide showing sucker of scolex as pointed by an arrow. (B) Histopathology slide showing hook of the scolex.

The histopathology slide showed the presence of suckers and hooks of the scolex which confirmed the diagnosis of cysticercosis cellulose. The sucker of the scolex can be seen in Figure 5 (A), as pointed by the arrow and Figure 5 (B) shows the hook of the sucker. The steroid was tapered over 1 month and glasses were prescribed as the Best Corrected Visual Acuity was 6/36. The patient followed up after 3 months and silicone oil was removed. The final BCVA was 6/12 in the right eye and the patient was well satisfied as her vision was preserved.

## DISCUSSION

Human cysticercosis occurs when man ingests the eggs of Taenia Solium. Cysticercosis cellulose is the larval stage of Taenia Solium. Thirty-five percent of the cysts in the eye are reported to occur in the subretinal space.^4^ High amplitude spikes are seen corresponding to cyst wall and scolex in Amplitude-scan. Hanging drop sign i.e. echoes corresponding to the cyst with the scolex attached to the inner wall is seen in Brightness can ultrasonography.^5^ Computed Tomography scan of the orbit is an effective technique to establish a diagnosis of ocular cysticercosis. It is fast and economical when compared to MRI. Cysticercosis is seen as a hypodense mass with a central hyper-dense scolex.^6^

Intraocular living cysticercosis will initiate a foreign body reaction, varying from mild uveitis to a panophthalmitis. Untreated cysticercosis will die and release toxins that induce an inflammatory reaction that may lead to the destruction of the eye. Hence, surgery is the treatment of choice in the treatment of posterior segment cysticercosis.^7^

Subretinal cysticercosis is treated by killing the organism in situ with drug therapy or removed surgically. The medical treatment is doubtful as the drugs used either do not cross the ocular barriers or penetrate the ocular barriers at non therapeutic doses. The organism should be carefully removed conserving its wall integrity to avoid the release of the intraocular antigen that causes a severe postoperative inflammatory response.^8^ The subretinal cyst in our case was surgically removed in a one-eyed patient that was vision-threatening. The cyst contents could spill and threaten the vision of the patient if vitrectomy was delayed in this challenging case. Steinmetz, et al. also reported successful removal of a subretinal cysticercus after fragmentation of the cyst inside the vitreous cavity by pars plana vitrectomy. The risk of spilling cyst contents is minimal if cutting or aspiration of the cyst is avoided which is similar in our case. The presence of a space between the cysticercus and the overlying retina, as demonstrated by our preoperative Amplitude Brightness (AB) scan ultrasonography also suggests the possibility of removing the cyst in toto similar to this case.^9^

The surgical removal of cyst in-toto was described in another study also to prevent inflammatory reaction and ocular damage. There was a good postoperative gain in visual acuity although the cysticercosis underneath the macula made the management challenging similar to our case. But in contrast to our study, they described the swept-source changes in optical coherence tomography too.^10^

The diagnosis in our case was made clinically by indirect ophthalmoscopic examination. A cyst was seen as a translucent white cyst with a dense white spot forming the invaginated scolex. The diagnosis of cysticercosis was confirmed by AB scan, MRI, and histopathological examination. After surgical removal, the cyst in toto was sent for histopathological examination. The scolex with suckers and a double row of hooklets is a prominent feature of cysticercosis cellulosae.^11^ The histopathology in our case showed the presence of scolex with hooklets which confirmed the diagnosis of Cysticercosis cellulosae.

The strength of our study is that this is a unique case of subretinal cysticercosis, follow up was regular and the surgical outcome was fruitful. The limitation is that the MRI and CT scan reports were missing from the patient although reporting was available. This case report highlights the importance of timely diagnosis and management of subretinal cysticercosis in a vision-threatening and challenging case. Subretinal cysticercosis occurs rarely, but the early diagnosis is very important to prevent the threatening of vision. Subretinal cyst can still be removed surgically in toto without spilling the contents even in a challenging case.
